# Comparison of continuous femoral nerve block (CFNB/SA) and continuous femoral nerve block with mini-dose spinal morphine (CFNB/SAMO) for postoperative analgesia after total knee arthroplasty (TKA): a randomized controlled study

**DOI:** 10.1186/s12871-016-0205-2

**Published:** 2016-07-16

**Authors:** Petchara Sundarathiti, Jadesadha Thammasakulsiri, Supawadee Supboon, Supalak Sakdanuwatwong, Molruedee Piangjai

**Affiliations:** Department Of Anesthesiology, Ramathibodi Hospital, Mahidol University, Bangkok, Thailand

**Keywords:** Continuous femoral nerve block (CFNB), Total knee arthroplasty (TKA), Levobupivacaine, Mini-dose spinal morphine, Postoperative analgesia

## Abstract

**Background:**

Unsatisfactory analgesia for major knee surgery with femoral nerve block (FNB) alone was reported and the additional benefit of sciatic block to continuous femoral nerve block (CFNB) was not conclusive. The aim of the present study was to find the benefit of the additional mini-dose spinal morphine (0.035 mg) to CFNB for postoperative pain control and to compare their associated side effects after total knee arthroplasty (TKA).

**Methods:**

After written informed consent and with Institutional Ethics Committee approval, 68 American Society of Anesthesiologists (ASA) Physical Status I-III patients scheduled for elective unilateral TKA under spinal anesthesia (SA) were included in the present prospective, randomized controlled study. The patients were allocated into two groups. CFNB was placed in all patients by the inguinal paravascular approach with 20 ml of 0.25 % levobupivacaine. Group I (named CFNB/SA group), SA was administered with 2.8 ml levobupivacaine and Group II (named CFNB/SAMO group), SA with 2.8 ml levobupivacaine plus morphine 0.035 mg. At Post Anesthesia Care Unit (PACU), pain and other adverse effects were recorded. Pain was assessed by visual analog scale (VAS) 0-10. Tramadol 50 mg intravenous (IV) was given if the VAS > 4. In the ward, all patients were maintained by continuous femoral infusion of 0.125 % levobupivacaine rate 7 ml/hr and then reduced to 5 ml/hr if VAS ≤3.

**Results:**

Patient’s demographics data in each group were not different. At post-operative (PO) 12-24 h, the VAS scores were significantly lesser in the CFNB/SAMO group. Cumulative tramadol IV requirement for PO48h were also significantly lesser in the CFNB/SAMO group. Nausea, vomiting and numbness were significantly greater in the CFNB/SAMO group during early postoperative period (PO1-6 h).

**Conclusion:**

Though in some patients CFNB was inadequate, a mini-dose of intrathecal morphine (0.035 mg) in addition to CFNB was found to be effective with minimal side effects.

**Trial registration:**

Thai Clinical Trial Registry (identifier: TCTR20150609003, date of registration: 6 June 2015).

## Background

A large number of patients who undergo knee surgery experience moderate to severe postoperative pain that interferes with participation in early physical therapy [1–3]. Severe postoperative pain can contribute to immobility-related complications, delay in hospital discharge, and interfere with functional outcome [4, 5]. Multiple techniques of postoperative pain control have been used after total knee arthroplasty (TKA). Previous studies comparing peripheral nerve block (PNB) with epidural analgesia (EA) for major knee surgery have demonstrated comparable analgesia and improvement in side-effect profile associated with PNB [6, 7].

The femoral nerve along with contributions from the sciatic and obturator nerves at the posterior and the medial aspects respectively, provide sensory innervations of the knee. These three terminal nerves are targeted by PNB techniques for major knee surgery [6, 8–10]. Reports of satisfactory analgesia with femoral nerve block (FNB) alone [2, 3] are countered by studies that found it to be inadequate [11–13]. Sundarathiti P et al. reported a lesser analgesia efficacy at postoperative (PO) 6-12 h in continuous FNB (CFNB) group compared with epidural analgesia [7]. Though sciatic blocks analgesic effect is undisputed, there are conflicting opinions about its general benefits in light of the additional time, costs and required skill of therapists [6, 10].

In response of surgeons’ concerns regarding postoperative sciatic block (e.g, difficulty in diagnosing peroneal nerve injury or an evolving sciatic nerve injury from compartment syndrome), we attempted to limit its use. The aim of the present study was to find the effect of adding a mini-dose spinal morphine 0.035 mg (based on our pilot study) to CFNB for postoperative pain control in patients with total knee arthroplasty (TKA).

### Ethical consideration

This study was conducted according to the declaration of Helsinki. Informed consent was obtained and documented from the participants before data collection. The final protocol and written informed consent form had been approved by Ethics Committee, Faculty of Medicine Ramathibodi Hospital, Mahidol University. This study has been registered at Thai Clinical Trial Registry (identifier: TCTR 20150609003).

## Methods

After written informed consent and with Institutional Ethics Committee approval from June 2012 to June 2015, 70 American Society of Anesthesiologists (ASA) physical status I-III patients scheduled for elective unilateral TKA under spinal anesthesia (SA) were included in the prospective, randomized controlled blind study. Exclusion criteria included age < 40 years or > 80 years, body mass index (BMI) > 45, renal insufficiency [Creatinine level (Cr) > 1.5 mg/dl], pre-existing neurological deficit, inability to comprehend pain scales, chronic opioid use, and contraindications to either neuraxial block or FNB. The patients were allocated into two parallel groups at a ratio of 1:1, using random number table. All patients were premedicated with oral lorazepam 0.5 mg 1 h before surgery and were sedated with midazolam 1 mg and fentanyl 25 mcg intravenously before conducting anesthesia. CFNB was placed in all patients by the inguinal paravascular approach, 19 G, 50 mm needle (PAJUNK®, PlexoLong NanoLine acc, Meier, Germany). Localized femoral nerve was defined by quadriceps twitch at <0.5 mAmp using a stimulation of 0.1 ms at 2 Hz. After negative aspiration, 20 ml of 0.25 % levobupivacaine was administrated and catheter was inserted 3-4 cm past the cannula. SA was done in lateral position at lumbar vertebrae segment (L) 3-4, 27-G needle. Group I (named CFNB/SA), received SA with 2.8 ml levobupivacaine. Group II (named CFNB/SAMO), received SA with 2.8 ml levobupivacaine plus morphine 0.035 mg. Urinary catheters were placed in all patients and were continued until 24 h post-operative.

The standard monitoring was used, including none invasive blood pressure, blood oxygen saturation (SpO_2_), electrocardiogram. The surgical time was noted as the time from incision to the end of surgery. On arrival in the Postanesthesia Care Unit (PACU), pain, and other adverse effects such as nausea, vomiting, pruritus, dizziness, hypotension (30 % reduced from baseline) numbness, and motor blockade were recorded every 15 min. Motor blockade was estimated using a modified Bromage scale (0 = no blockade: extended limb lift off the bed; 1 = flexion/extension at knee and ankle joint; 2 = no flexion/extension at knee or ankle joint; 3 = complete blockade). Pain was assessed by visual analog scale (VAS) from 0-10, where 0 = no pain; 1-3 = mild pain; 4-7 moderate pain; 8-10 = severe pain. Tramadol 50 mg intravenous (IV) was given if the VAS ≥ 4.

Forty eight hours post-operative in the ward, patients in both groups were maintained by continuous infusion of 0.125 % levobupivacaine rate 7 ml/hr and then reduced to 5 ml/hr if VAS ≤3. The femoral catheters were removed at 48 h post-operative. During the hospital stay, all patients received oral ultracet one tab two times a day, oral acetaminophen 500 mg four times a day, and oral lorazepam 0.5 mg before bed time. The patients having breakthrough pain (defined as VAS ≥4) were treated on demand with tramadol 50 mg IV every 4 h until discharge. The blinded residents made visits at 6, 12, 24, 36, and 48 h post-operative to record adverse effects, pain scores, patients’ satisfaction (1 = poor, 2 = fair, 3 = good, and 4 = excellent).

**Fig. 1:**
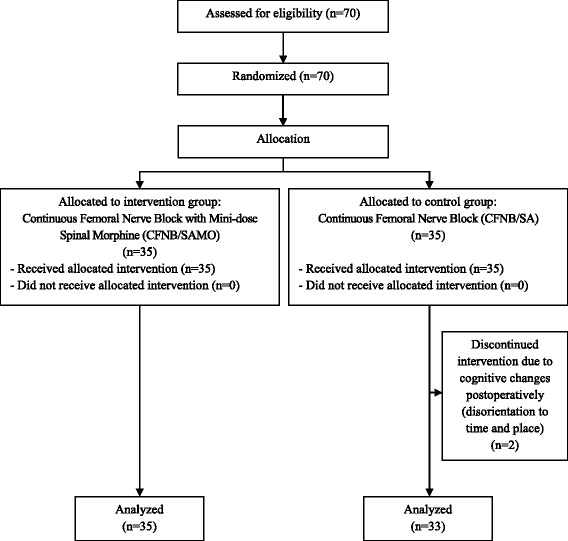
CONSORT 2010 Flow diagram

Based on the data from Sundarathiti et al. [7], at 12 h post-operative, 80 % of patients with CFNB after TKA experienced moderate to severe pain, while approximately 38 % of patients with continuous epidural infusion (CEI) did. About 22 patient in each group would suffice to demonstrate a significant difference with a probability of type I error of 0.05 and power value of 80 %. The long-term expected rate of patient loss was considered to be about 20 %, so finally each group was to have at least 26 patients Detailed information on enrollment of patients into the study is depicted by the CONSORT flow diagram in Fig. 1.

Data were recorded and analyzed using SPSS 15 statistical package (SPSS; Inc., Chicago, IL) for Windows. Results are reported as mean (SD) for continuous variables, where number and percentage for nominal variables. All continuous variables were first checked for normality of data distribution by Shapiro-Wilk test. Independent samples *T*-test or Mann–Whitney *U* test were used to compare the data between the two study groups depending on data distribution in each measurement. Nominal variables were analyzed by chi-square or Fisher’s exact test. A p-value < 0.05 was considered statistically significant.

## Results

A total of 68 patients participated, 33 with spinal anesthesia and femoral catheter, and 35 with the same procedure plus intrathecal morphine. Patient’s demographics data (Table 1) in each group were not different. All patients had satisfactory anesthesia and operation without intaoperative complications. There were 2 patients in Group I (CFNB/SA) dropped out from the study due to cognitive changes postoperatively (disorientation to time and place). Residual motor blockade was similar in both group (Table 2).

**Table 1:** Demographic data

	CFNB/SA	CFNB/SAMO	*p*-value
	(*n* = 33)	(*n* = 35)	
Age (yr)	68.06 (8.37)	69.60 (7.39)	0.42
Weight (kg)	59.88 (14.42)	62.00 (12.72)	0.52
Height (cm)	154.76 (6.19)	155.86 (6.94)	0.49
Body mass index (BMI) (kg/m^2^)	26.39 (5.61)	25.74 (4.49)	0.60
ASA (I/II/III)	0/13/20	1/7/27	0.11
Surgical time (mins)	155.30 (34.41)	145.86 (39.06)	0.30

Data are expressed as number of patients or mean (SD); *CFNB/SA* Continuous femoral nerve block/Spinal anesthesia; *CFNB/SAMO* Continuous femoral nerve block/Spinal anesthesia plus Morphine

**Table 2:** Modified Bromage scale (MBS) at 0, 15, 30, 45 and 60 min

MBS	0 min		15 min		30 min		45 min		60 min	
	CFNB/SA	CFNB/SAMO	CFNB/SA	CFNB/SAMO	CFNB/SA	CFNB/SAMO	CFNB/SA	CFNB/SAMO	CFNB/SA	CFNB/SAMO
	(*n* = 33)	(*n* = 35)	(*n* = 33)	(*n* = 35)	(*n* = 33)	(*n* = 35)	(*n* = 33)	(*n* = 35)	(*n* = 33)	(*n* = 35)
0	-	-	-	-	-	-	-	-	-	-
1	-	-	2 (6.06)	-	3 (9.09)	1 (2.86)	5 (15.15)	5 (14.29)	7 (21.21)	10 (28.57)
2	7 (21.21)	8 (22.86)	8 (24.24)	16 (45.71)	12 (36.36)	24 (68.57)	20 (60.61)	27 (77.14)	22 (66.67)	24 (68.57)
3	26 (78.79)	27 (77.14)	23 (69.70)	19 (54.29)	18 (54.55)	10 (28.57)	18 (24.24)	3 (8.57)	4 (12.12)	1 (2.86)
*p*-value	0.87		0.29		0.10		0.27		0.23	

The values are expressed as number of patients (percent)

*MBS* modified bromage scale (0 = no blockade; 1 = for flexion/extension at knee and ankle joint; 2 = no flexion/extension at knee and ankle joint; and 3 = complete blockade)

**Fig. 2:**
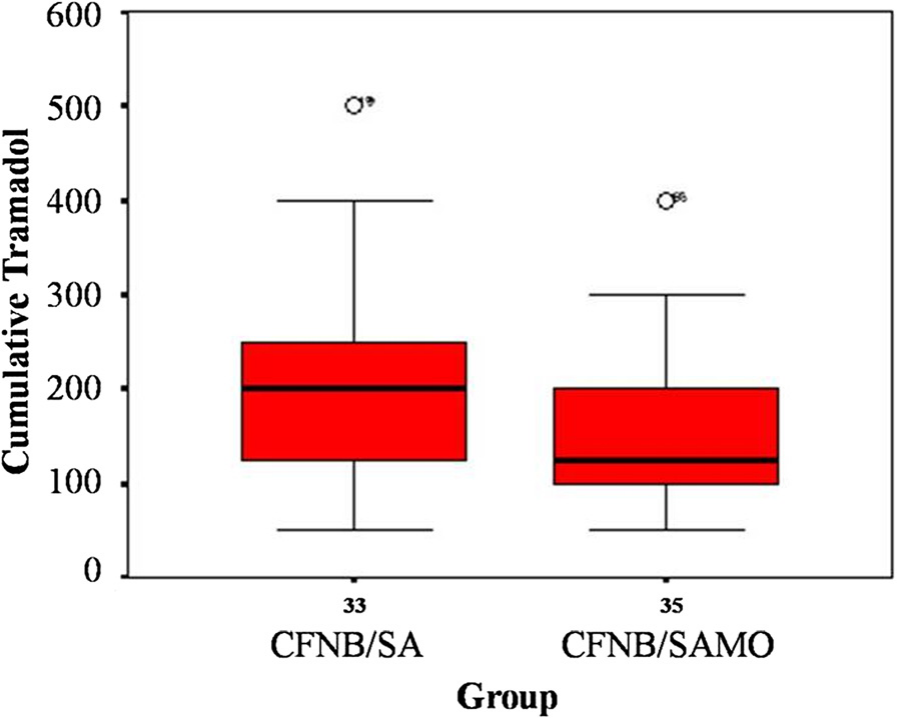
Cumulative tramadol IV requirement for PO48h

**Fig. 3:**
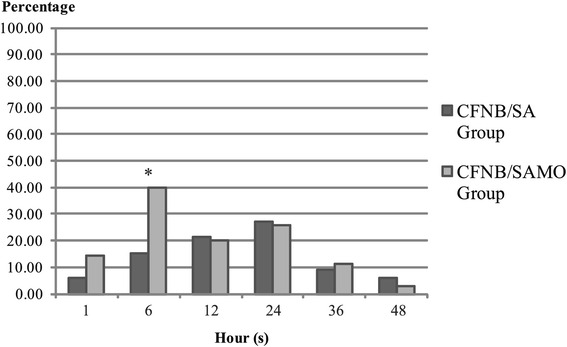
Incidence of nausea/vomiting

The VAS scores and the number of patients suffering from ‘moderate to severe pain’ are presented in Tables 3 and 4. Experiencing pain was significantly less pronounced in patients with spinal morphine (CFNB/SAMO), starting 6 h after surgery during the entire investigation period; these differences were significant at 6, 12, 24, and 48 h respectively. Cumulative tramadol IV requirement was significantly lesser in the CFNB/SAMO group (median = 125 mg, range 50-400 mg) compared with the CFNB/SA group (median = 200 mg, range 50-500 mg) as presented in Fig. 2 (*p*-value = 0.01). As presented in Fig. 3, at 6 h after surgery a significant higher rate of CFNB/SAMO patients were affected by PONV (approx. 40 vs. 15 %; *p* < 0.05). Patients in both groups had similar overall incidences of other side effect such as pruritus, dizziness and hypotension (*p*-value >0.99 in all side effects) except the incidences of numbness during PO1-6 h which was significantly greater in the CFNB/SAMO group (p-value 0.03 and 0.04 respectively). Patient’s satisfaction rated as good or excellent was not different between the two groups.

**Table 3:** Median (range) of VAS scores

	CFNB/SA	CFNB/SAMO	*p*-value
	(*n* = 33)	(*n* = 35)	
Postoperative hr	VAS (1 – 10)		
1	0 (0-2)	0 (constant)	0.14
6	5 (0-10)	3 (0-9)	0.05
12	6 (0-10)	3 (0-7)	<0.01
24	5 (0-10)	3 (0-7)	<0.01
36	3 (0-10)	2 (0-8)	0.18
48	4 (0-10)	3 (0-7)	0.03

Data are shown as median (min-max)

**Table 4:** Incidence of moderate to severe pain (VAS 4-7)

	CFNB/SA	CFNB/SAMO	*p*-value
	(*n* = 33)	(*n* = 35)	
Postoperative, hr	Patients, n (%)		
1	-	-	-
6	22 (66.67 %)	17 (48.57 %)	0.13
12	23 (69.70 %)	15 (42.86 %)	0.03
24	22 (66.67 %)	16 (45.71 %)	0.08
36	13 (39.39 %)	11 (31.43 %)	0.49
48	17 (51.52 %)	9 (25.71 %)	0.03

Data are shown as number and percentage

## Discussion

In this study, we used a very small dose of intrathecal morphine 0.035 mg in addition to femoral block (CFNB/SAMO) to complete the analgesia without any more effort and care on the side of the anesthesiologists. The pain scores (VAS) 6, 12, 24 and 48 h after surgery and the cumulative tramadol IV requirement (Fig. 2) were significantly lesser in the CFNB/SAMO group compared with the CFNB/SA group. The VAS scores at PO1-6 h were not different, which may be partly due to the residual analgesia after spinal anesthesia. Whereas motoric function was not affected by the respective method, the rate of PONV was signifivantly higher in patients with morphine at the 6^th^ hour after surgery (approx. 40 vs. 15 %) but not any further (Fig. 3).

The effect of CFNB in this study was not satisfactory, because the VAS scores in both groups were quite high (Table 3). Categorizing VAS scores ≥ 4 as ‘failure’ we observed a failure rate between 6 and 48 h postoperatively of 39.4 – 69.7 % and 31.4 – 48.6 % in patients with CFNB and patients with CFNB plus spinal morphine respectively (Table 4). Pöpping et al.^9^ in their large observational study including 1374 patients with femoral/sciatic block had a failure rate (no analgesic effect) of 3.96 % and a rate of malposition after correct placement of 15.2 %. Among others we have to contemplate the possibility of secondary block failure, where the catheter has not been positioned appropriately. Although the block was achieved with the stimulation technique, the trigger during stimulation applied in this study with < 0.5 mA was probably too high. In addition the initial dose of local anesthetics might have been too low and unnoticed catheter migration could also lead to failure. Inadequate efficacy of FNB alone for knee surgery is due to the fact that the posterior part of the knee is innervated by the sciatic nerve. Hence sciatic block has been added to FNB by many study groups [8, 9, 12–14], though it is unclear whether blocking both the femoral and sciatic nerve may result in a greater risk of direct needle trauma to the peripheral nerve. Performance of sciatic nerve block is time-consuming, requiring additional costs and skill. However, our data clearly indicate that femoral block alone is not sufficient in major knee surgery, as it does not affect the popliteal area, which is in accordance to Sundarathiti P et al. [7] reporting inferior analgesic efficacy of FNB compared with epidural analgesia.

The purpose of our study was to find a simple ‘multimodal’ form of postoperative pain control, using a technique that can be added to femoral catheter in patients with major knee surgery under spinal anesthesia. Multimodal analgesia combines alternative strategies with the goal to avoid routine parenteral narcotics, and minimize the side effects of the respective method [15–18]. It takes advantage of the synergistic effects of various analgesics, permitting the use of smaller doses.

Intrathecal opioids added to local anesthetics during spinal anesthesia have been applied in a variety of surgical settings since 1979 [19], providing prolonged postoperative analgesia without the need for catheters or expensive pumps. However, the use of intrathecal morphine may be associated with distressing side effects, such as itching, urinary retention, nausea and vomiting (PONV), and respiratory depression [20]. In an attempt to limit opioid side effects, the use of low-dose spinal opioids has been advocated [21]. Even mini-dose morphine (<0.1 mg) was frequently reported to be effective for managing acute postoperative pain after variety of surgeries without any evidence of respiratory depression [22, 23].

Achieving high quality pain relief after TKA is possible using regional anesthesia and multimodal pain management [24]. In patients already treated with spinal anesthesia intrathecal opioid analgesia (ITOA) has specific advantages regarding ease of administration, safe and rapid onset of action and low costs. A single dose administered at the time of surgery can provide good neuroaxial analgesia during the first postoperative day. Because spinal morphine is frequently accompanied by a high rate of PONV and itching, we tried a very low dose with 0.035 mg and found a temporary increase in numbness which was clinically irrelevant, but a significant increase in PONV 6 h after surgery. There were no further or late side effects. We can state mini-dose intrathecal morphine (ITMO) is a safe, effective, and relatively inexpensive modality for the management of postoperative pain. We estimate CFNB and mini-dose ITMO as a good combination-technique to achieve the goal of multimodal pain management for TKA.

## Conclusion

Postoperative analgesia with continuous femoral block (CFNB) alone for total knee arthroplasty (TKA) is inadequate. A mini-dose of intrathecal morphine in addition to CFNB was found to be simple, safe, inexpensive and more effective than CFNB alone for managing postoperative pain after TKA with little side effects. However, the analgesic effect could be better. Future studies have to determine the most appropriate dose of spinal morphine when added to proper working femoral analgesia.

## Abbreviations

ASA, American Society of Anesthesiologists; BMI, body mass index; CFNB, continuous femoral nerve block; CFNB/SAMO, continuous femoral nerve block with mini-dose spinal morphine; Cr, creatinine level; EA, epidural analgesia; ITMO, intrathecal morphine; ITOA, intrathecal opioid analgesia; IV, intravenous; PACU, post anesthesia care unit; PNB, peripheral nerve block; PO, post-operative; PONV, post-operative nausea and vomiting; SA, spinal anesthesia; TKA, total knee arthroplasty; VAS, visual analog scale.
